# Early home literacy and math environment: cross-domain associations between parental literacy and math beliefs

**DOI:** 10.3389/fpsyg.2025.1649713

**Published:** 2025-08-28

**Authors:** Jamlick Peter Ondieki Bosire, Francisco Palermo, Amy R. Napoli

**Affiliations:** ^1^Department of Human Development and Family Science, University of Missouri, Missouri, MO, United States; ^2^Department of Child, Youth and Family Studies, University of Nebraska-Lincoln, Lincoln, NE, United States

**Keywords:** home math, home literacy, home learning environment, parental beliefs, parental expectations, parental math anxiety, appropriateness of activities, preschool children

## Abstract

We examined how parents’ beliefs are related to home environment practices within and across academic domains (i.e., literacy and math) and the mechanisms through which parents’ beliefs shape home environments. The sample included 945 parents of 0–6-year-old children (*M* = 4.01, *SD* = 1.55) from the Early Home Learning Environment dataset. Parents answered questions on beliefs, expectations, perceived appropriateness of activities, math anxiety, and home literacy (HLE) and math (HME) learning environment activities. Using path analysis, we examined the extent to which parents’ beliefs, expectations, perceptions of activity appropriateness, and math anxiety were associated with the HLE and HME; whether expectations for children mediate the associations between parental beliefs and home environments; and whether perceptions of appropriateness of activities and math anxiety moderate the association between parental beliefs, expectations, and home environments. Literacy expectations and perceived appropriateness of literacy activities were positively associated with the HLE, while literacy beliefs and perceptions of appropriateness of literacy activities were associated with the HME. Math beliefs, expectations, and perceptions of appropriateness of math activities were positively associated with the HME, whereas math beliefs, perceptions of appropriateness of math activities, and math anxiety were associated with the HLE. Expectations were only significant within domains and mediated the associations between beliefs and activities for both literacy and math. Only perceptions of appropriateness of math activities moderated the association between math beliefs and HLE. Our study supports the interconnectedness of HLE and HME activities and the importance of parental beliefs in shaping home learning environments.

## 1 Introduction

Language and literacy skills are deeply connected with early math skills and are influenced by similar factors, such as the home learning environment and caregivers’ language use (Anders et al., 2012; Davidse et al., 2014; Manolitsis et al., 2013; Purpura et al., 2011). Further, the co-occurrence of language, literacy, and math disorders is common, with estimates suggesting that they overlap in 30–70% of cases (Moll et al., 2019). Research has consistently emphasized the importance of home literacy (HLE) and math (HME) environments in supporting children’s language, literacy, and math skills (Daucourt et al., 2021; Sénéchal et al., 2017). Central to the home learning environment are parents’ beliefs, which shape how parents engage with their children and view their abilities, as well as the kinds of resources and opportunities that they provide children at home (Elliott et al., 2021; Napoli et al., 2021). Existing research has examined how parents’ beliefs influence within-domain home environments (i.e., how literacy beliefs relate to HLE activities and how math beliefs relate to HME activities)—often treating them as separate and distinct. However, considering the interrelatedness of language, literacy, and math skills (Moll et al., 2019; Purpura et al., 2011), there is a need to investigate how parents’ beliefs relate to home learning activities across domains and the possible mechanisms through which beliefs relate to activities. This will aid in developing more targeted and effective early learning environments and may help the field to understand the intricacies of the home learning environment and help parents optimize learning opportunities at home. Thus, in this study, we explored how parents’ beliefs about literacy and math relate to home environment activities within and across both domains. We also examined the mediating role of parental expectations for children and the moderating roles of perceptions of appropriateness of activities and parental math anxiety.

### 1.1 Home learning environment

Two primary aspects of the home learning environment are the home literacy and math environments (Napoli and Purpura, 2018; Salminen et al., 2021). Both environments present contexts where young children develop and acquire language, literacy, and math skills (Sénéchal, 2006; Skwarchuk et al., 2014). Parents support these skills in several ways, including providing children with appropriate learning materials and resources, engaging in warm and responsive interactions with them devoid of hostility, modeling learning through stimulating activities like shared reading and problem solving, and ensuring the physical environment is calm and uncluttered to encourage children to interact and explore safely (Caldwell and Bradley, 1984; Korucu and Schmitt, 2020; Rodriguez and Tamis-LeMonda, 2011; Son and Morrison, 2010).

#### 1.1.1 Home literacy environment (HLE)

The HLE includes aspects of the home environment that support the development of children’s language and literacy skills (Foster et al., 2005). Seminal home literacy models organize the HLE into oral language and code-related (i.e., text decoding) activities (Sénéchal, 2006; Sénéchal and LeFevre, 2002). Oral language activities (e.g., reciting the alphabet, rhymes) support children’s oral language skills, and code-related activities (e.g., parent teaching of letters) support children’s code skills. Oral language skills help children to process the *meaning* of verbal and written language (e.g., vocabulary), and code-related skills help children to understand the *code* of written language (e.g., grapheme-phoneme correspondence). Several studies have linked the HLE with language, literacy, and math skills (e.g., Anders et al., 2012; Manolitsis et al., 2013; Riordan et al., 2018). For example, shared book reading—which often integrates both oral and code-related elements—is associated with children’s expressive vocabulary, emergent literacy skills, language comprehension, and even early math abilities (Hindman et al., 2014; Purpura et al., 2021; Riordan et al., 2018).

In contrast, less supportive HLEs are associated with weaker vocabulary, early reading skills, and overall school readiness. These environments generally lack consistent engagement in enriching literacy activities and thus provide limited opportunities for learning. For instance, children in homes with limited shared reading experiences, few print-rich materials, and minimal opportunities for meaningful, responsive parent–child interactions often exhibit smaller vocabularies and weaker decoding and phonological awareness skills—foundational components of early reading (Rodriguez and Tamis-LeMonda, 2011; Son and Morrison, 2010). Moreover, infrequent conversations, storytelling, or exposure to diverse language use can hinder children’s ability to comprehend texts and express themselves effectively. These disadvantages accumulate over time, placing children at a disadvantage when they enter formal schooling.

#### 1.1.2 Home math environment (HME)

The HME comprises the characteristics of the home environment that support the development of children’s early math skills (Hornburg et al., 2021; Skwarchuk et al., 2014). The HME includes numeracy, geometry, spatial, and measurement activities (Ehrman et al., 2023; Milburn et al., 2019; Zippert and Rittle-Johnson, 2020). These activities can be introduced to children informally (e.g., playing games that involve counting) or formally (e.g., teaching counting). Formal (i.e., structured activities with a clear learning goal) and informal (i.e., learning opportunities embedded in everyday routines and play) activities benefit children’s development of non-symbolic and symbolic math skills (Skwarchuk et al., 2014). Several studies have linked HME activities to children’s language, literacy, and mathematical skills (e.g., Daucourt et al., 2021; LeFevre et al., 2009; Manolitsis et al., 2013; Mutaf-Yıldız et al., 2020; Vandermaas-Peeler and Pittard, 2014).

Conversely, limited exposure to math-related activities and conversations at home has been linked to lower early math achievement. An unsupportive HME – characterized by infrequent caregiver use of numerical language, scarce opportunities for counting, comparing quantities, or measuring, and a general absence of math engagement – may hinder the development of early math skills (Manolitsis et al., 2013; Mutaf-Yıldız et al., 2020). For instance, DeFlorio and Beliakoff (2015) found that children from homes with minimal math talk generally performed worse on math assessments. Without regular, meaningful math experiences embedded in daily routines – such as discussing shapes, estimating quantities, or playing number-based games – children may miss key opportunities to develop foundational math skills, including number sense, counting, and spatial reasoning. Over time, such experiences contribute to disparities in school readiness and sustained differences in math achievement throughout the early years (Skwarchuk et al., 2014).

### 1.2 Cross-domain association of math, language, and literacy

A growing body of research highlights the interconnected development of early language, literacy, and mathematical skills. Although these domains have traditionally been studied separately, increasing evidence suggests that foundational language and literacy abilities—such as print knowledge, vocabulary, rhyming, letter recognition, emergent writing, and orthographic knowledge—play a vital role in shaping early mathematical competencies (Davidse et al., 2014; Purpura et al., 2011). Language is a cognitive and communicative tool, supporting children’s understanding of and engagement with mathematical concepts. For example, vocabulary knowledge helps children comprehend math-specific terms (e.g., more, less, equal, difference) and interpret word problems (Hornburg et al., 2024). Additionally, syntax and sentence comprehension are linked with mathematical understanding, enabling children to follow multi-step instructions and grasp the structure of math tasks (Chow and Ekholm, 2019). Oral language skills also facilitate children’s ability to explain their reasoning and strategies during problem-solving (Purpura et al., 2011).

Recent findings also suggest that mathematical language may predict later reading skills and mediate early math and literacy development (Purpura et al., 2017). Some studies suggest that early mathematical skills may be stronger predictors of later literacy than early language or literacy skills alone (Claessens and Engel, 2013; Napoli and Purpura, 2018). This may be due to the cognitive demands of math activities, which often require symbolic representation, pattern recognition, sequencing, and logical reasoning—skills that are also foundational for narrative comprehension and other literacy-related tasks. Consistent with these shared underpinnings, comorbidity among language, literacy, and math disorders is common, with estimates suggesting that they co-occur in approximately 30–70% of cases (e.g., Moll et al., 2019). The high overlap further supports the need for a cross-domain perspective in early academic research and intervention.

Beyond these cognitive connections, shared environmental factors—such as parental beliefs, home learning activities, and the language caregivers use during interaction or instruction—have been linked to both domains (Anders et al., 2012; Korucu and Schmitt, 2020; Manolitsis et al., 2013; Rodriguez and Tamis-LeMonda, 2011). Parents who frequently engage children in rich verbal and literacy-related interactions may also incorporate numeracy-related content through everyday routines like counting, measuring, or problem-solving (Manolitsis et al., 2013; Purpura et al., 2011, 2017). These overlapping environmental and cognitive associations highlight the importance of adopting a cross-domain perspective when examining early learning. Building on this perspective, the current study examines whether parents’ literacy and math beliefs are associated with domain-specific home practices and cross-domain environments, contributing to a more integrated understanding of how early learning is shaped within the home.

### 1.3 Parental beliefs and the home learning environment

Parents’ engagement in children’s activities at home is not arbitrary—it is shaped by their underlying beliefs about their perceived responsibility in child rearing, expectations about child development, and the perceptions of confidence in supporting children’s learning and development (Hoover-Dempsey et al., 2005). These beliefs, expectations, and perceptions impact the frequency and nature of parent–child interactions in the home learning environment (Elliott et al., 2021; LeFevre et al., 2010; Slicker et al., 2021). Situated expectancy-value theory (Eccles and Wigfield, 2020) explains that parents are more likely to participate in activities they see as valuable and where they hold high expectations for their children’s success. In other words, when parents perceive a learning activity as beneficial for their child’s development, they are more inclined to engage with it. This theory offers a comprehensive model for understanding how beliefs about the importance of an activity and the expectation of success influence parents’ motivation and engagement.

Examining how parental beliefs relate to the home learning environment involves considering the pathways through which these beliefs manifest in everyday routines. Parental beliefs may correspond with expectations for children’s academic readiness, which in turn may be reflected in how parents organize and prioritize learning experiences at home (Del Río et al., 2017; Martini and Sénéchal, 2012; Varnell et al., 2025). For example, a parent who believes a child should know how to read by kindergarten entry may be more inclined to engage in literacy-focused interactions. However, other psychological factors, such as perceptions of the developmental appropriateness of activities and math-related anxiety, may influence how beliefs and expectations are enacted (Del Río et al., 2017; Guzmán et al., 2023). Even when two parents report similar expectations for early math learning, the parent who perceives math activities as more appropriate or feels less anxious about math may be more likely to provide math learning opportunities. These interrelated beliefs and attitudes may shape the home learning environment in nuanced ways across domains.

Parental expectations refer to beliefs about the knowledge and skills children should acquire at particular ages (Martini and Sénéchal, 2012). These expectations often reflect what parents view as important for school success and can vary across domains (e.g., literacy vs. math). Previous research has linked these expectations to variation in parental involvement in early literacy and numeracy activities with their children (Del Río et al., 2017; Martini and Sénéchal, 2012). For instance, parents who expect their children to master reading or basic math by school entry may report more structured academic engagement at home. Yet, expectations do not necessarily predict behavior uniformly. Parental math anxiety—characterized by discomfort, tension, or worry related to math—may correspond with reduced confidence or willingness to engage in math activities, regardless of high expectations (Del Río et al., 2017; Guzmán et al., 2023). Similarly, parents’ beliefs about whether specific academic tasks are age-appropriate may influence the timing and frequency of those activities. For example, counting or letter-writing may be postponed if viewed as developmentally premature, even among parents with high educational aspirations (Hoover-Dempsey et al., 2005; Slicker et al., 2021). Together, these cognitive and psychological processes interact in ways that may shape how families support early learning across literacy and math domains.

Sociodemographic factors also appear to be associated with variation in parental beliefs about early learning. For instance, Zheng and Libertus (2018) found that parents with higher levels of education were more likely to emphasize formal math exposure, while those with higher income placed greater value on fostering literacy activities in the home. Other studies suggest that parents with low income may be more likely than parents with higher income to view preschools as primary settings for academic learning, possibly reflecting lower self-efficacy regarding their role in home-based instruction (Bosire et al., 2024; Drummond and Stipek, 2004). These patterns highlight the complexity of parental belief systems and point to the importance of understanding how various factors intersect to shape early learning experiences in the home.

### 1.4 Cross-domain associations of parental beliefs

Ample research has examined domain-specific associations between parental beliefs and home learning environments. Studies have found that parents’ beliefs about literacy are associated with literacy-related home activities (e.g., Elliott et al., 2021) while beliefs about math are associated with math-focused home activities (e.g., Napoli et al., 2021). However, some findings are mixed. Napoli et al. (2021) reported that while parents’ numeracy beliefs were positively associated with HME, literacy beliefs were not significantly linked to HLE. In contrast, other studies (e.g., Bergman Deitcher et al., 2024; Elliott et al., 2021; Wu and Hindman, 2025) found associations between literacy and numeracy beliefs and their corresponding home environments. Some studies suggest that parents tend to prioritize literacy over math and feel more confident supporting literacy activities (Blevins-Knabe et al., 2000; Cannon and Ginsburg, 2008; Napoli et al., 2021; Warren and Young, 2002), while others, such as Elliott et al. (2021) suggest that parents view both math and literacy as equally important areas of learning.

Although these domain-specific approaches have advanced our understanding of how beliefs shape home practices, they may overlook possible cross-domain associations—for example, whether beliefs in one academic area are related to behaviors in another. This gap is particularly relevant given increasing evidence that language and literacy skills are closely interconnected with mathematical development, and vice versa (Purpura et al., 2011, 2017; Hornburg et al., 2024). If early learning domains are developmentally intertwined, it is plausible that parents’ beliefs could reflect or support patterns of engagement that span multiple domains. For example, a parent with strong expectations regarding their child’s literacy development might demonstrate similar levels of structure, encouragement, and involvement in math-related activities, even if their math-specific beliefs differ. Supporting this possibility, Elliott et al. (2021) found that parents who rated early literacy skills as highly important also reported greater engagement in basic numeracy activities.

Despite the plausibility of these cross-domain links, empirical research examining such associations is limited. The present study addresses this gap by examining whether parental beliefs about literacy and math are independently associated with the HLE and HME. By taking a cross-domain perspective, this study provides a more integrated understanding of how parental beliefs may relate to multiple dimensions of the early home learning environment.

### 1.5 Current study

The current study examined the within-and cross-domain associations between parental beliefs and HLE and HME activities. We also examined the mechanisms through which parental beliefs are associated with HLE and HME activities by examining whether parental expectations mediate the associations between parental beliefs and activities, and whether parents’ perceptions of appropriateness of activities and math anxiety moderate the associations between parental expectations and activities, and parental beliefs and activities. Understanding how parental beliefs in one domain relate to the other may help develop targeted, holistic interventions to support parents in fostering rich home environments. Three research questions guided this study:

1. How are parents’ literacy and math beliefs, expectations, math anxiety, and perceptions of appropriateness of activities related to HLE and HME activities?

*Hypothesis 1 (H1)*: Parents’ literacy and math beliefs, expectations, math anxiety, and perceptions of the appropriateness of activities will each be significantly associated with their engagement in home literacy (HLE) and home math (HME) activities.

2. Do parents’ expectations for children mediate the associations between parental beliefs and HLE and HME activities?

*Hypothesis 2 (H2)*: Parents’ expectations for their children’s literacy and math development will mediate the associations between their corresponding beliefs and their engagement in HLE and HME activities.

3. Do parents’ perceptions of appropriateness of math and literacy activities and math anxiety moderate the association between parental beliefs, expectations, and activities?

*Hypothesis 3 (H3)*: Parents’ perceptions of the appropriateness of literacy and math activities and their math anxiety will moderate the mediated pathways proposed in H2, such that the indirect effects will be stronger at higher levels of perceived appropriateness and lower levels of math anxiety.

## 2 Materials and methods

### 2.1 Procedure

We used publicly available data from the Early Home Learning Environment dataset (EHLE; Ellis et al., 2022a). These cross-sectional data were collected between December 2021 and January 2022 via Prolific^1^, an online platform for recruiting research participants. To be eligible, participants had to be 18–75 years old, U. S. citizens living in the United States, have at least one child, and speak English as their first language (Ellis et al., 2022b). Participants were instructed to complete the survey based on their youngest child, born between 2015 and 2021 (i.e., aged 0–13). The participants were informed that the survey would include various questions regarding their child’s home learning environment, and they received compensation of $8.90 per hour.

### 2.2 Participants

The EHLE dataset includes 1,046 U. S.-based parents of children aged 0 to 13 years. We focused on a subset of the data consisting of parents who reported their child’s age as between 0 and 6 years because we were interested in the early home learning environment. We also excluded 52 parents who did not report on their home learning environment. The final sample included 945 parents, predominantly of middle-upper socioeconomic status, whose ages ranged from 18 to 64 years with 50.9% of parents being between 25 and 34 years old. On average, children were 4.01 years old (*SD* = 1.55); there were 31 children less than 1 year old, 40 one-year-olds, 67 two-year-olds, 207 three-year-olds, 187 four-year-olds, 235 five-year-olds, and 178 six-year-olds. See Table 1 for additional parent and child demographic information.

**Table 1:** Demographic information and descriptive statistics.

Variable	Descriptive statistics	
	*N*	%
Child’s sex		
Male	465	49.2
Female	477	50.5
Non-binary/third gender	2	0.2
Other	1	0.1
Parent race/ethnicity		
White, non-Hispanic	813	86
Black, non-Hispanic	66	7
Asian	20	2.1
American Indian or Alaska Native	4	0.4
Native Hawaiian or Pacific Islander	2	0.2
Middle Eastern or North African	1	0.1
Other	6	0.6
Bi/Multi-Racial	33	3.5
Parent gender		
Male	296	31.3
Female	636	67.3
Non-binary/third gender	12	1.3
Other	1	0.1
Household income		
Less than $10,000	29	3.1
$10,001 to $19,999	29	3.1
$20,000 to $29,999	54	5.7
$30,000 to $39,999	85	9
$40,000 to $49,999	82	8.7
$50,000 to $59,999	93	9.8
$60,000 to $69,999	75	7.9
$75,000 to $79,999	98	10.4
$80,000 to $89,999	65	6.9
$90,000 to $99,999	55	5.8
$100,000 to $149,999	179	18.9
$150,000 or more	101	10.7
Parent education level		
Some high school	9	1
High school graduate or GED	87	9.2
Some college	198	21
2-year degree (e.g., AA, AS)	106	11.2
4-year degree (e.g., BA, BS)	330	34.9
Graduate degree (e.g., MS, MA, MSW, Med)	180	19
Doctorate (e.g., PhD, JD, EdD, DDS)	35	3.7
Parent relationship with child		
Mother	637	67.4
Father	299	31.6
Other	9	1

### 2.3 Measures

All the data were collected via a Qualtrics survey.

#### 2.3.1 Parental beliefs

Parents responded to three items capturing math beliefs (*α* = 0.85) and two items capturing literacy beliefs (*α* = 0.67; Zippert and Rittle-Johnson, 2020). Parents rated the importance of their child doing well in various activities using a 5-point Likert scale—*not at all important* (1), *slightly important* (2), *important* (3), *fairly important* (4), and *very important* (5). The math activities included counting, comparing, and naming numbers; building with blocks and doing puzzles; and noticing and making patterns. Literacy activities included learning to read and write and talking with others. The items were summed by scale so that higher scores indicated higher beliefs, with a possible range of 3–15 for math and 2–10 for literacy.

#### 2.3.2 Parental expectations

Parents responded to four items capturing math expectations (*α* = 0.83) and four items capturing literacy expectations (*α* = 0.88; LeFevre et al., 2009). Parents rated the importance of their child doing well in various activities prior to kindergarten entry using a 5-point Likert scale—*not at all important* (1), *of little importance* (2), *of average importance* (3), *important* (4), and *very important* (5). The math activities included children counting to 10, counting to 100, identifying/recognizing written numbers, and knowing simple sums. Literacy activities included the children rehearsing the alphabet, identifying/recognizing alphabet letters, printing their name, and printing alphabet letters. The items were summed by scale so that higher scores indicate higher expectations for a possible range of 4–20 for math and 4–20 for literacy.

#### 2.3.3 Appropriateness of activities

To assess the perceived appropriateness of activities, parents responded to 60 math items (*α* = 0.97) adapted or drawn from existing measures (e.g., Mutaf-Yıldız et al., 2020; Napoli and Purpura, 2018; Purpura et al., 2020; Zippert et al., 2020) and 13 literacy items (*α* = 0.87) drawn from McCormick et al. (2020). Parents rated the activities using a 3-point Likert scale: *too easy* (1), *just right* (2), and *too hard* (3). Sample math activities included counting objects, printing numbers, playing board games, drawing maps, comparing sizes of numbers, identifying patterns, and using scales. Sample literacy activities included writing letters, rhyming, retelling/making up stories, and naming objects. The items were summed so that higher scores indicated higher perceived appropriateness of activities for a possible range of 69–180 for math and 13–39 for literacy.

#### 2.3.4 Parent math anxiety

Parents responded to 14 items (*α* = 0.94) adopted from Cosso et al. (2021) assessing their math anxiety. Parents reported if they felt anxious when asked to do various math activities using a 5-point Likert scale: *not true of me at all* (1), *generally not true of me* (2), *somewhat true of me* (3), *generally true of me* (4), and *very true of me* (5). Sample math activities included being asked to add 976 and 777, calculating a tip without a calculator, looking at math text, reading a number book, and solving a math-related riddle. The items were summed so that higher scores indicate higher levels of math anxiety for a possible range of 14–70.

#### 2.3.5 Home learning activities

Items measuring HME activities were adapted from established tools in prior research (e.g., Mutaf-Yıldız et al., 2020; Napoli and Purpura, 2018; Purpura et al., 2020; Zippert et al., 2020) and covered the four HME domains (i.e., numeracy, spatial/geometry, patterning, and measurement). Items measuring HLE activities were from McCormick et al. (2020) and covered oral language and code domains. HME activities comprised 60 items (*α* = 0.96) and HLE activities comprised 13 items (*α* = 0.89), all asking parents to report how often they engaged in the activities with their child. Responses were given on a 6-point Likert scale: *never* (1), *1–3 times a month* (2), *about once a week* (3), *2–5 times per week* (4), *daily* (5), and *multiple times a day* (6). Sample HME activities included counting objects, reading number storybooks, identifying names of written numbers, reading number stories, playing card games, noting numbers on signs when walking/driving, interacting with clocks, using a computer for spatial games, using kits to draw models, talking about math in reference to sports, and discussing patterns. Sample HLE activities included showing a child how to read a book, practicing writing letters, practicing sounding letters and rhyming learning, having the child explain part of a storybook, teaching about the world around them, reading books, and naming objects in books. The items were summed and higher scores indicated higher frequency, ranging from 60 to 346 for HME and 13–78 for HLE activities.

#### 2.3.6 Control Variables

We included the child’s age, parents’ educational level, and household income as covariates because existing studies have established that they are associated with parents’ beliefs and children’s home learning environment (Elliott et al., 2021; Napoli et al., 2021).

### 2.4 Analytic plan

All data analyses were done using SPSS 29 and Mplus 8.4 (Muthén and Muthén, 2019). Collinearity diagnostics were conducted prior to analysis, including examining variance inflation factors (VIFs) and tolerance values. The results indicated that multicollinearity was not a concern, as all VIFs were below the commonly accepted threshold of 5, and tolerance values were within acceptable ranges. We conducted a preliminary analysis to compute descriptive statistics and examine bivariate correlations. The full-information maximum likelihood procedure (FIML) was used to handle cases with missing data. To answer the first research question of within-and cross-domain associations, we used path analysis by modeling HME and HLE on parental math beliefs, math expectations, appropriateness of math activities, parental literacy beliefs, literacy expectations, and appropriateness of literacy activities. We controlled for children’s age, parental education, and household income by testing direct paths from those variables to significant covariances.

To answer the second research question, examining whether parental expectation mediated the association between parental beliefs and HLE and HME activities, we examined indirect effects using bootstrapping (Shrout and Bolger, 2002), allowing for the empirical estimation of the sampling distribution of indirect effects. The 95% confidence intervals (CIs) for the indirect effect estimates were calculated using a non-parametric bootstrapping method with 500 resamples to generate bias-corrected CIs.

**Figure 1:**
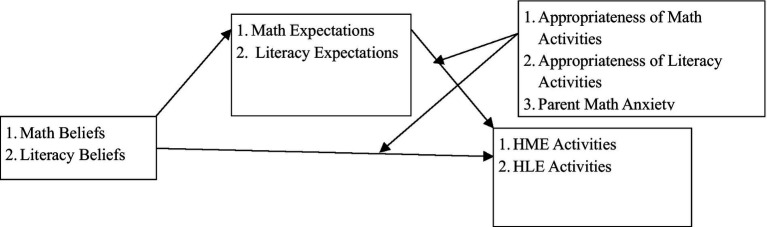
Hypothesized mechanisms through which parental beliefs shape home learning environments.

To answer the third research question, we tested a moderated mediation model using path analysis to examine how parental beliefs related to HLE and HME activities, whether parental expectation acted as a mediator in those associations, and whether perceptions of appropriateness of activities and math anxiety moderated the mediated pathways (see Figure 1 for our conceptual model). All variables were standardized (*M* = 0, *SD* = 1), and eight interaction terms were created to test moderation effects. Four examined moderations of mediated paths: (1) parental numeracy expectations × perceptions of appropriateness of numeracy, (2) parental literacy expectations × perceptions of appropriateness of literacy, (3) parental numeracy expectations × parental math anxiety, and (4) parental literacy expectations × parental math anxiety. The other four examined the moderation of direct paths: (1) parental numeracy beliefs × perceptions of appropriateness of numeracy, (2) parental literacy beliefs × perceptions of appropriateness of literacy, (3) parental numeracy beliefs × parental math anxiety, and (4) parental literacy beliefs × parental math anxiety. Model fit was evaluated based on the Comparative Fit Index (CFI; values above 0.90 indicate an acceptable fit), the Root Mean Square Error of Approximation (RMSEA; values below 0.06 indicate good fit), and the Standardized Root Mean Square Residual (SRMR; values below 0.08 indicate good fit; Bentler, 1990; Hu and Bentler, 1999).

## 3 Results

### 3.1 Preliminary results

**Figure 2:**
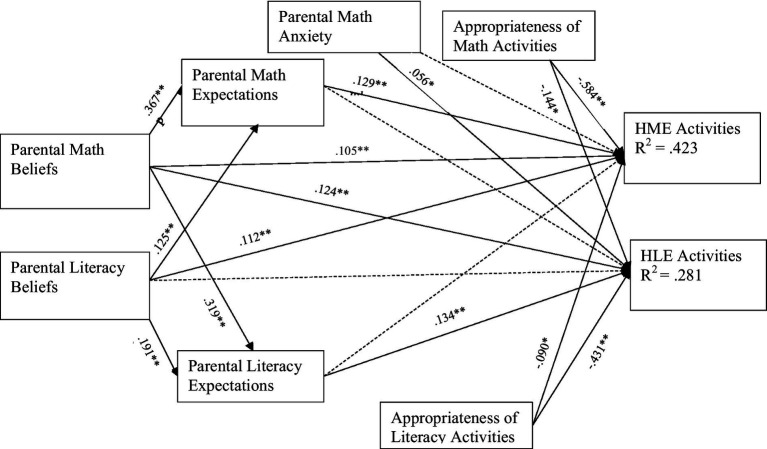
Associations among parental beliefs, expectations, appropriateness of activities, and HME and HLE activities. We controlled for child age, parent education, and household income. The dotted paths represent non-significant associations (CFI = 0.938, RMSEA = 0.051, 95% CI [0.050, 0.067], SRMR = 0.053). *p < 0.05, **p < 0.01.

See Table 2 for the bivariate correlations. Table 3 includes all of the parameter estimates for the effects tested in our path model depicted in Figure 2. As shown in Table 3, parental math beliefs were positively and significantly associated with parental math expectations. Relatedly, parental math anxiety was negatively associated with parental math expectations. Children’s age was negatively associated with parental math expectations for children. The cross-domain associations revealed that parental literacy beliefs were positively associated with parental math expectations. However, parents’ education and income were not significantly associated with parents’ math expectations for children.

**Table 2:** Bivariate correlations of the study variables.

Variable	1	2	3	4	5	6	7	8	9
1. Math beliefs	–								
2. Literacy beliefs	0.572^**^	–							
3. Math expectations	0.449^**^	0.339^**^	–						
4. Literacy expectations	0.434^**^	0.381^**^	0.770^**^	–					
5. Appropriateness of math activities	−0.116^**^	0.059	−0.112^**^	−0.107^**^	–				
6. Appropriateness of literacy activities	−0.085^**^	−0.047	−0.104^**^	−0.129^**^	0.752^**^	–			
7. HME activities	0.188^**^	0.059	0.220^**^	0.182^**^	−0.631^**^	−0.511^**^	–		
8. HLE activities	0.180^**^	0.080^*^	0.153^**^	0.199^**^	−0.411^**^	−0.496^**^	0.678^**^	–	
9. Parental Math Anxiety	−0.131**	−0.055	−0.128**	−0.094**	0.160**	0.120**	0.014	0.171	
*M*	12.26	9.21	3.48	3.83	136.98	27.31	154.77	52.17	2.07
*SD*	2.55	1.33	0.88	0.94	20.40	4.35	45.22	12.99	0.92

*p < 0.0.05, **p < 0.0.01; HLE, home literacy environment; HME, home math environment.

**Table 3:** Standardized coefficients for covariates, direct, indirect, and moderated tested in Figure 2.

Path	*β*	S. E	Est./S. E	*p*	Confidence Intervals (CI)—low/high
Covariates					
Parents’ education → HLE activities	0.029	0.036	0.825	0.409	−0.037/0.103
Household income → HLE activities	0.029	0.036	0.828	0.408	−0.037/0.098
Children’s age (years) → HLE activities	−0.097	0.048	−2.015	0.044	−0.189/−0.005
Parents’ education → HME activities	−0.026	0.034	−0.748	0.455	−0.094/0.047
Household income → HME activities	−0.014	0.032	−0.426	0.670	−0.117/0.043
Children’s age (years) → HME activities	−0.042	0.041	−1.041	0.298	−0.032/0.064
Parents’ education → Parental literacy expectation	0.038	0.035	1.087	0.277	−0.029/0.109
Household income → Parental literacy expectation	0.088	0.036	2.450	0.014	0.058/0.485
Children’s age (years) → Parental literacy expectation	−0.006	0.029	−0.209	0.835	−0.018/0.157
Parents’ education → Parental math expectation	0.047	0.038	1.226	0.220	−0.063/0.047
Household income → Parental math expectation	0.026	0.038	0.692	0.489	−0.163/0.296
Children’s age (years) → Parental math expectation	−0.062	0.030	−2.030	0.022	−0.067/−0.002
Within-domain effects					
Parental literacy expectations → HLE activities	0.134	0.048	2.829	0.005	0.046/0.230
Parental literacy beliefs → HLE activities	−0.064	0.034	−1.864	0.062	−0.130/0.009
Perceptions of literacy appropriateness → HLE activities	−0.431	0.052	−8.250	<0.001	−0.521/−0.315
Parental literacy beliefs → Parental literacy expectations	0.191	0.038	5.038	<0.001	0.116/0.264
Parental numeracy expectations → HME activities	0.129	0.039	3.303	0.001	0.059/0.207
Parental numeracy beliefs → HME activities	0.105	0.033	3.169	<0.001	0.035/0.165
Perceptions of numeracy appropriateness → HME activities	−0.584	0.051	−11.471	<0.001	−0.688/−0.481
Parental math anxiety → HME activities	0.031	0.026	1.178	0.239	−0.022/0.079
Parental numeracy beliefs → Parental math expectations	0.367	0.035	10.351	<0.001	0.295/0.437
Parental math anxiety → Parental math expectations	−0.062	0.030	−2.030	0.042	−0.126/−0.006
Cross-domain effects					
Parental numeracy expectation → HLE activities	−0.051	0.046	−1.101	0.267	−0.132/0.034
Parental numeracy beliefs → HLE activities	0.124	0.037	3.393	0.001	0.052/0.192
Perceptions of numeracy appropriateness → HLE activities	−0.144	0.058	−2.496	0.013	−0.264/−0.035
Parental math anxiety → HLE activities	0.056	0.028	1.969	0.049	0.000/0.114
Parental numeracy beliefs → Parental literacy expectations	0.318	0.037	8.522	<0.001	0.244/0.388
Parental math anxiety → Parental literacy expectations	−0.011	0.032	−0.354	0.723	−0.078/0.054
Parental literacy expectations → HME activities	0.004	0.038	0.104	0.917	−0.071/0.077
Parental literacy beliefs → HME activities	−0.078	0.029	−2.708	0.007	−0.137/−0.024
Perceptions of literacy appropriateness → HME activities	−0.090	0.045	−2.002	0.045	−0.174/−0.029
Parental literacy beliefs → Parental math expectations	0.125	0.036	3.473	0.001	0.054/0.193
Mediational effects					
Parental literacy beliefs → Parental literacy expectations → HLE activities	0.023	0.011	2.158	0.031	0.008/0.050
Parental literacy beliefs → Parental math expectations → HLE activities	−0.005	0.006	−0.734	0.463	−0.020/0.004
Parental math beliefs → Parental math expectations → HME activities	0.044	0.015	3.004	0.003	0.021/0.077
Parental math beliefs → Parental literacy expectations → HME activities	−0.003	0.012	−0.220	0.826	−0.024/0.025
Moderated effects					
Parental literacy expectations × Perceptions of literacy appropriateness → HLE activities	0.087	0.049	1.757	0.079	−0.010/0.186
Parental literacy expectations × Parental math anxiety → HLE activities	−0.031	0.050	−0.620	0.535	−0.133/0.066
Parental literacy beliefs × Perceptions of literacy appropriateness → HLE activities	−0.021	0.038	−0.560	0.576	−0.101/0.051
Parental literacy beliefs × Parental math anxiety → HLE activities	0.008	0.035	0.241	0.809	−0.054/0.076
Parental math expectation ×Perceptions of math appropriateness → HLE activities	−0.062	0.047	−1.323	0.186	−0.155/0.028
Parental math expectations × Parental math anxiety → HLE activities	0.020	0.051	0.402	0.688	−0.073/0.119
Parental math beliefs × Parental math anxiety → HLE activities	−0.027	0.032	−0.845	0.398	−0.095/0.038
Parental math beliefs × Perceptions of math appropriateness → HLE activities	0.082	0.037	2.198	0.028	0.005/0.154
Parental literacy expectations × Perceptions of literacy appropriateness → HME activities	0.044	0.040	1.115	0.265	−0.027/0.124
Parental literacy expectations × Parental math anxiety → HME activities	−0.073	0.041	−1.761	0.078	−0.150/0.016
Parental literacy beliefs × Perceptions of literacy appropriateness → HME activities	−0.030	0.031	−0.950	0.342	−0.089/0.027
Parental literacy beliefs × Parental math anxiety → HME activities	−0.022	0.028	−0.802	0.423	−0.075/0.033
Parental math expectation ×Perceptions of math appropriateness → HME activities	−0.074	0.045	−1.663	0.096	−0.166/0.003
Parental math expectations × Parental math anxiety → HME activities	0.027	0.041	0.656	0.512	−0.054/0.102
Parental math beliefs × Parental math anxiety → HME activities	0.006	0.028	0.223	0.824	−0.048/0.060
Parental math beliefs × Perceptions of math appropriateness → HME activities	0.037	0.034	1.104	0.270	−0.030/0.101
R-square					
Parental math expectations	0.216	0.025	8.455	<0.001	
Parental literacy expectations	0.221	0.027	8.068	<0.001	
HLE activities	0.281	0.029	9.785	<0.001	
HME activities	0.423	0.029	14.855	<0.001	

Parental literacy beliefs were positively associated with parental literacy expectations. Household income was positively associated with parental literacy expectations. Children’s age and parents’ education were not significantly associated with parental literacy expectations. Cross-domain associations showed that parental math beliefs were positively associated with parental literacy expectations. Parental math anxiety was not significantly associated with parental literacy expectations.

### 3.2 How are math and literacy beliefs, expectations, math anxiety, and perceptions of activity appropriateness related to HLE and HME activities?

The within-domain associations revealed that parental math beliefs and math expectations were positively associated with HME activities (see Table 3). Perceptions of appropriateness of math activities were negatively associated with HME activities. Parental math anxiety was not significantly associated with HME activities. The covariate effects of children’s age, parents’ education, and household income were not statistically significant.

Parental literacy expectations were positively associated with HLE activities. The perceptions of appropriateness of literacy activities were negatively associated with HLE activities. Parental literacy beliefs were not significantly associated with HLE activities. Children’s age was significantly and negatively associated with HLE activities but parents’ education and income were not significantly associated.

Cross-domain associations revealed that parental literacy beliefs and perceptions of appropriateness of literacy activities were negatively associated with HME activities. Parental literacy expectations were not associated with HME activities. Parental math beliefs and parental math anxiety were positively associated with HLE activities. The perceptions of appropriateness of math activities were significantly and negatively associated with HLE activities. Parental math expectations for children were not significantly associated with HLE activities.

### 3.3 Do parents’ expectations for children mediate the associations between parental beliefs and HLE and HME activities?

Parental math beliefs were positively associated with HME activities through parental math expectations but not through parental literacy expectations (see Table 3). Similarly, parental literacy beliefs were positively associated with HLE activities through parental literacy expectations but not through parental math expectations.

### 3.4 Do parents’ perceptions of appropriateness of math and literacy activities and math anxiety moderate the association between parental beliefs, expectations, and HLE and HME activities?

**Figure 3:**
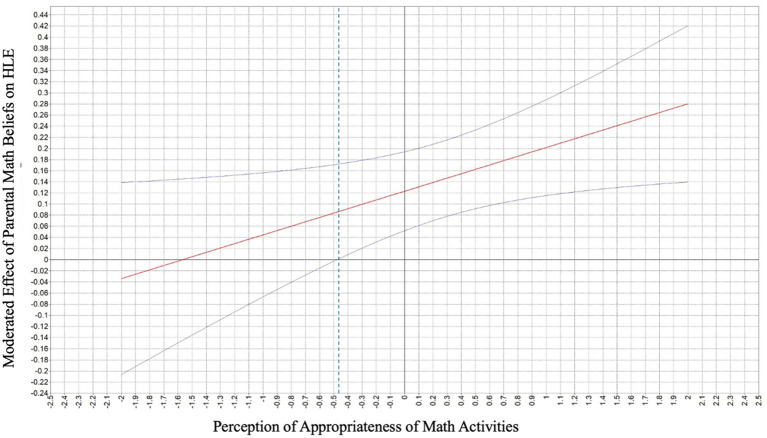
The moderating effect of perception of appropriateness of math activities on parents’ math beliefs on HLE activities. The upper and lower curved lines are the 95% confidence intervals around the adjusted effect of perception of appropriateness of math activities on the association between parental math beliefs and HLE activities. HLE, home literacy environment.

None of the interaction terms (i.e., within-or cross-domain) were associated with HME activities (see Table 3). For HLE activities, only parental math beliefs × perceptions of appropriateness of math activities was associated with HLE activities. We performed simple slope analyses at (+/− 1 *SD*) above and below the adjusted effect of perception of appropriateness of math activities on the association between parental math beliefs and HLE activities and the Johnson–Neyman technique to visualize the moderated effect along with its 95% confidence intervals across the full range of parental perceptions of appropriateness of math activities (Hayes, 2018; Johnson and Neyman, 1936). The plot in Figure 3 shows how the association between parental math beliefs and HLE activities changes depending on how appropriately parents perceived the math activities. The solid line represents the strength (slope) of this association across varying levels of perceived appropriateness of math activities. The dashed vertical line marks the point where the confidence interval for this effect includes zero, meaning the association is no longer significant. According to the plot, this point occurs at about −0.45 standard deviations below the mean of perceived appropriateness of math activities (i.e., 127.8 for the unstandardized variable), implying that for parents who view math activities as highly inappropriate (−0.45 *SD* below average), the influence of their beliefs about math on HLE is not significant. However, for parents with increased perception of the appropriateness of math activities, their beliefs about math matter in supporting more HLE activities.

## 4 Discussion

We investigated how parents’ beliefs in specific domains (i.e., math or literacy) relate to their home environment practices both within and across domains, whether parental expectations for their children mediated these associations, and whether their perceptions of activity appropriateness and parental math anxiety moderated those associations. The hypotheses were partially supported. Within domains, parental math beliefs and expectations positively associated with HME activities, while parental literacy expectations—but not beliefs—were positively linked to HLE activities. Cross-domain associations showed that literacy beliefs were negatively associated with HME activities, whereas math beliefs were positively associated with HLE activities. Mediation analyses confirmed that parental expectations mediated within-domain associations. Additionally, a significant interaction between perceptions of appropriateness of math activities and parental math beliefs moderated the association between math beliefs and HLE activities.

### 4.1 Within-domain associations

#### 4.1.1 Math

Parental math beliefs and parental math expectations for children were positively associated with HME activities. These findings align with other studies suggesting that parental math beliefs directly relate to HME activities, and parental expectations positively relate to HME activities (e.g., Del Río et al., 2017; Napoli et al., 2021; Varnell et al., 2025). This evidence stresses the critical role of parents’ beliefs about math and the need to foster in parents’ higher expectations for their children’s math skills to promote quality HME interactions (Liu and Chung, 2022). Interestingly, perceptions of appropriateness of math activities were negatively associated with HME activities. Varnell et al. (2025) found that although parents perceived math activities as equally appropriate for boys and girls, patterns in their actual engagement evolved over time; after 4 years, parents reported engaging more frequently in math activities with boys than with girls. The current study adds an important layer to this finding—parents who view math activities as less appropriate for their children’s development may reduce engagement in such activities even when they hold strong beliefs and expectations for the children in the math domain. This implies that perceptions of appropriateness of math activities—whether accurate or not—serve as a gatekeeper and are associated with differences in how HME is structured. Interventionists working to enhance the HME can help parents assess their children’s developmental readiness.

Contrary to earlier studies (e.g., Del Río et al., 2017), which found that parental math anxiety was negatively related to engagement in math activities, our findings suggest a more complex association. Specifically, parental math anxiety was not significantly associated with HME activities. Instead, it was indirectly associated with the HME through math beliefs, expectations, and perceptions of appropriateness of math activities, which were linked to HME activities. Higher math anxiety was associated with lower math beliefs and expectations for children, yet it also correlated with higher perceptions of appropriateness of math activities. This may reflect cognitive dissonance among mathematically anxious parents—while they may view math activities as appropriate for their children, they may lack confidence in their ability to support these activities, leading to lower math-related beliefs and expectations (Guzmán et al., 2023). This may explain the lack of a direct association between parental math anxiety and HME activities. The finding aligns with recent research characterizing parental math anxiety as a multidimensional construct (Cosso et al., 2021), and it extends the literature by highlighting potential pathways through which math anxiety may be associated with the HME.

#### 4.1.2 Literacy

Parental literacy expectation was significantly and positively associated with HLE activities, whereas literacy beliefs were not. This pattern is consistent with studies that have found maternal literacy expectations to be related to the frequency of formal and informal literacy engagement, while literacy beliefs did not show the same association (Liu and Chung, 2022; Napoli et al., 2021). However, other research has identified links between literacy beliefs and HLE activities (e.g., Bergman Deitcher et al., 2024; Elliott et al., 2021; Wu and Hindman, 2025). Variations in these findings may stem from differences in sample characteristics, measurement tools, or contextual factors such as cultural and socioeconomic background. The current results indicate that stronger parental expectations around literacy often co-occur with more frequent literacy-related activities at home. In contrast, positive beliefs about literacy may not always align with reported behaviors (Liu et al., 2018). Consistent with our findings on the HME, parental perceptions of the appropriateness of literacy activities were negatively associated with HLE activities. Few studies have explored the role of perceptions of appropriateness of literacy activities in shaping HLE activities, so more research is needed to corroborate this finding. Investigating whether targeting perceptions of appropriateness of literacy activities in interventions could improve HLE may be a valuable next step. Together, these findings highlight a meaningful association between parental expectations and perceptions of appropriateness of literacy activities and the nature of the HLE.

Notably, while parental literacy beliefs were positively correlated with literacy expectations, they were negatively associated with parents’ perceptions of the appropriateness of literacy activities. This may imply that though parental literacy beliefs may be associated with higher literacy expectations, those beliefs may not necessarily correspond with greater engagement—particularly when accompanied by doubts about the appropriateness of literacy activities (Stephenson et al., 2008). Evidence of this disconnect comes from a study showing that mothers tended to engage children in literacy activities more than fathers, despite similarly high literacy expectations reported by both fathers and mothers (Liu and Chung, 2022). However, the study by Liu and Chung did not measure the appropriateness of activities.

Finally, we found that children’s age was significantly and negatively associated with HLE activities, suggesting that as children grow older, parents engage them in fewer literacy activities. This is potentially due to shifting perceptions of child independence (Sénéchal and LeFevre, 2002). Another possible explanation is the inclusion of 6-year-olds in the current study, as parental engagement may shift when children enter kindergarten. In contrast, Son and Morrison (2010) focused on children aged 36–54 months and observed a general increase in HLE activities as children grew older.

### 4.2 Cross-domain associations

#### 4.2.1 Beliefs

The findings highlight the complex, interconnected nature of parental beliefs across math and literacy domains and how these beliefs align with distinct patterns of engagement in the home learning environment. Parental literacy beliefs were significantly and negatively associated with HME activities, suggesting that parents with strong beliefs about literacy may reduce their engagement in math activities. This aligns with prior research indicating that when parents hold higher beliefs in literacy skills, they tend to emphasize basic math activities that can be easily integrated into literacy activities and avoid the more structured, math-centered activities (Elliott et al., 2021). These patterns may be linked to practical considerations such as limited time and resources. As such, the home learning environment appears to reflect not only the types of activities that take place, but also the priorities parents place on them. To capture this complexity of the home learning environment, researchers should examine HLE and HME activities as distinct domains, while also considering parents’ beliefs.

In contrast, parental math beliefs were significantly and positively associated with HLE activities, suggesting that stronger math beliefs accompany higher engagement in literacy activities. One possible explanation involves the cognitive overlap between math and literacy skills or a reflection of a broader orientation toward early academic preparedness. Tasks such as understanding numbers, solving word problems, and recognizing patterns often rely on narrative comprehension, strong language skills, and sequencing abilities—skills commonly nurtured through reading and storytelling (Davidse et al., 2014; Purpura et al., 2011). This highlights the importance of examining math and literacy domains independently and in relation to each other, with attention to parental beliefs. This approach may allow for a more nuanced and accurate understanding of how the home learning environment is structured and where differences in early learning opportunities may be observed.

#### 4.2.2 Expectations

Despite the substantial overlap between math and literacy skills and their comorbidity (Anders et al., 2012; Moll et al., 2019; Napoli and Purpura, 2018), neither parental literacy expectations nor parental math expectations were associated with learning activities across domains. This suggests that parents’ expectations are highly domain-specific and may not generalize from one domain to another. According to Expectancy-Value Theory (Eccles and Wigfield, 2020), expectancies for success are tied to specific tasks and domains, and individuals are more likely to engage in behaviors they believe their efforts will meaningfully influence. In this context, parents’ expectations reflect goal-directed aspirations, which likely motivate investment of time, resources, and effort into domain-specific activities.

Our findings align with this theoretical perspective: higher parental expectations in math were associated with richer HME activities, and higher literacy expectations were linked to more HLE activities—both within domain. This pattern supports the view that parents are more likely to engage in home learning behaviors that reflect their expectations for their child’s success in a specific area. At the same time, the lack of cross-domain associations may reflect a form of prioritization or resource allocation, whereby parents focus their engagement in the domain they deem more important or in which they feel more confident. Similar results have been observed in prior research, where literacy expectations did not predict early math outcomes (e.g., Segers et al., 2015).

These findings highlight how expectations may shape parental decision-making and resource distribution in the home learning environment, reinforcing the Expectancy-Value framework. Further research is needed to examine how these domain-specific expectations are formed and whether interventions can help broaden parents’ engagement across the domains.

#### 4.2.3 Perception of appropriateness of activities

Perceptions of developmental appropriateness were closely aligned with domain-specific patterns of engagement in the home learning environment. Specifically, perceptions of appropriateness of literacy activities were significantly and negatively associated with HME activities. This aligns with previous research indicating that literacy is often emphasized over mathematics in early childhood, reflecting commonly held views about developmental readiness (Blevins-Knabe et al., 2000; Cannon and Ginsburg, 2008; Warren and Young, 2002). Conversely, parental perceptions of the appropriateness of math activities were negatively associated with HLE activities. These patterns may be associated with practical considerations such as limited time, available resources, or varying levels of parental confidence, along with differing interpretations of what activities are most relevant for a child’s developmental stage. The findings highlight the relevance of examining cross-domain dynamics within the home learning environment. Exploring how engagement in one domain corresponds with patterns of engagement in another may offer a more comprehensive understanding of how early learning opportunities are distributed across domains within families.

#### 4.2.4 Math anxiety

In line with the cognitive overlap discussed earlier, the observed positive association between parental math anxiety and HLE activities may suggest a pattern of compensatory mechanism, where parents who are anxious about math may attempt to offset their discomfort by prioritizing literacy-related activities in the home. This pattern is consistent with previous research showing that parents with lower math anxiety tend to engage more in advanced numeracy activities with their children (Del Río et al., 2017). Rather than disengaging from their child’s learning, these parents may actively support early development through literacy practices that feel more accessible or less threatening. Another possible explanation for these patterns may relate to how parents perceive the academic nature of each domain. Parents may view math as a more formal, school-based subject requiring specialized instruction, making them more likely to defer responsibility to teachers. In contrast, literacy activities—such as shared book reading—are heavily promoted through public health campaigns, parenting programs, and children’s media. This may foster greater parental confidence and a sense of responsibility in supporting literacy at home. These findings point to the relevance of considering psychological factors and broader sociocultural influences when examining how families navigate early learning across domains.

### 4.3 Mediating role of parental expectations

The mediation analyses uncovered important insights into the mechanisms through which parental beliefs relate to HLE and HME activities. Parental math and literacy beliefs were positively associated with HME and HLE activities through parental math and literacy expectations. These findings align with studies emphasizing the role of parents’ expectations in shaping home learning environments (Del Río et al., 2017; Martini and Sénéchal, 2012; Slicker et al., 2021) and extend the literature by revealing the indirect paths through which parental beliefs may relate to home learning activities. Previous studies found that while parental math beliefs were directly associated with HME activities, parental literacy beliefs were not associated with HLE activities (e.g., Napoli et al., 2021). In the current study, parental expectations were statistically associated with the relation between parental literacy beliefs and HLE activities and the relation between math beliefs and HME activities—fully accounting for the former and partially for the latter. These findings suggest that parents who hold stronger math and literacy beliefs are more likely to set higher expectations in math and literacy for their children, which may foster their engagement in math and literacy activities at home (Segers et al., 2015; Slicker et al., 2021). Other studies have found that parent teaching at home is associated with parental expectations and that expectations explain unique variance in the HLE after controlling for child and family background characteristics (Martini and Sénéchal, 2012). No cross-domain mediation was found in the current study, further reiterating the earlier discussion that parents’ expectations in one domain may not significantly associate with engagement in another domain.

Interestingly, only household income was associated positively with parents’ literacy expectations, and children’s age was associated negatively with parents’ math expectations. This implies that parents with higher income levels may have higher literacy expectations for their children, and as children grow older, the parents tend to lower their math expectations. Other studies have found that parental engagement tends to decline as children grow older, and that higher parental income and education are associated with increased exposure to reading, writing, and active literacy activities at home; however, math activities appear to be unrelated to socio-economic status (Elliott et al., 2021; LeFevre et al., 2010; Zheng and Libertus, 2018). Taken together with the role of math anxiety, these findings may help explain the greater value placed on and higher engagement in literacy activities compared to math activities, as reported in previous studies (e.g., Blevins-Knabe et al., 2000; Cannon and Ginsburg, 2008; Napoli et al., 2021). Parents may view literacy and math as requiring distinct approaches or strategies. The differences in engagement may reflect varying levels of knowledge, familiarity, or comfort in supporting children’s learning across domains. These findings suggest that domain-specific parental expectations may be linked with how families engage in literacy and math activities at home. As such, efforts to support home learning may benefit from considering how expectations differ across literacy and math domains. Interventions that align with these domain-specific expectations could be more relevant and effective.

### 4.4 Moderating role of appropriateness of activities and math anxiety

None of the interaction terms, whether within-or cross-domain, were significantly associated with HME activities. This suggests that parental beliefs, expectations, math anxiety, and perceptions of appropriateness of activities are primarily associated with HME activities through direct associations. Stated differently, these factors appear to be independently associated with HME activities in additive rather than interactive ways, reflecting independent contributions of each construct to patterns of math engagement in the home. For the HLE, one significant interaction emerged—the interaction between parental math beliefs and perceptions of appropriateness of math activities was significantly associated with HLE activities. This cross-domain interaction suggests a nuanced relation across domains. Specifically, this finding suggests that how parents’ math beliefs relate to their engagement in HLE activities depends on how appropriate they perceive math activities to be for their child’s developmental stage. For example, stronger math beliefs may be associated with higher literacy engagement when parents view math activities as developmentally appropriate. Alternatively, when parents view math activities as less appropriate, strong math beliefs may not be associated with increased HLE activities. This pattern may reflect the interconnectedness of the HME and HLE (Elliott et al., 2021). Such findings point to the importance of perceived developmental appropriateness of math activities and beliefs as distinct but interrelated factors associated with HLE activities (Stephenson et al., 2008).

### 4.5 Implications

These findings have several practical implications for supporting early childhood learning at home. First, parent education programs should be tailored to address math anxiety and build parental confidence in engaging with early math alongside literacy. Providing parents with accessible, developmentally appropriate math activities can empower them to incorporate math into everyday routines more comfortably. Second, creating cross-domain learning resources that integrate literacy and math concepts may help parents recognize the interconnectedness of these skills and facilitate more holistic support at home. Third, given the diversity of family backgrounds, it is essential that interventions and resources are culturally sensitive, respecting varied parental beliefs and expectations while promoting engagement in both domains. Fourth, early identification of parental math anxiety or low confidence could allow for timely, targeted support, potentially mitigating challenges before children enter formal schooling. Finally, early childhood education policies and family engagement initiatives must ensure that support strategies account for the emotional and cognitive factors influencing parental involvement and are accessible to diverse populations. Addressing these areas may contribute to the creation of more effective home learning environments that foster children’s development across both literacy and math domains.

### 4.6 Limitations

There are several limitations in this study that should be acknowledged. The data were solely based on parental reports, hence, could be subject to bias. Parents’ responses may have reflected perceived expectations rather than their actual behaviors and beliefs. Future research should incorporate data triangulation through observations, questionnaires, and interviews. Secondly, the list of HLE and HME items used in the study does not fully encompass the range of home enrichment activities that families engage in. Future research should include a more comprehensive set of activities. Third, because we used a cross-sectional design, causal interpretations cannot be inferred. Finally, because the sample primarily consisted of families from middle to upper socioeconomic backgrounds, the findings may not be fully generalizable to more diverse or lower-SES populations. Despite these limitations, this study offers a fresh perspective on the associations between parents’ beliefs and the structure of the home learning environment.

### 4.7 Conclusion

This study illustrates the nuanced and multifaceted associations among parental beliefs, expectations, perceptions of appropriateness of activities, and math anxiety within home learning environments, with domain-specific expectations closely linked to patterns of engagement. While these factors show primarily direct associations with the HME, the observed interaction between math beliefs and perceptions of the appropriateness of math activities in relation to HLE activities reflects the interconnected nature of math and literacy practices in the home.

## Data Availability

Publicly available datasets were analyzed in this study. This data can be found: https://ldbase.org/datasets/2d78d002-9e32-44b0-b8ca-8e0f2291bed9.

## Footnotes

^1^ https://www.prolific.com
